# Supplementary material for: Amphotericin B-Silver Hybrid Nanoparticles Help to Unveil the Mechanism of Biological Activity of the Antibiotic: Disintegration of Cell Membranes
Source: Molecules. 2023 Jun 10;28(12):4687. doi: 10.3390/molecules28124687 (PMC10301372; doi:10.3390/molecules28124687)
Supplement: Supplementary file 1 [file molecules-28-04687-s001.zip › molecules-2430418-supplementary.pdf]

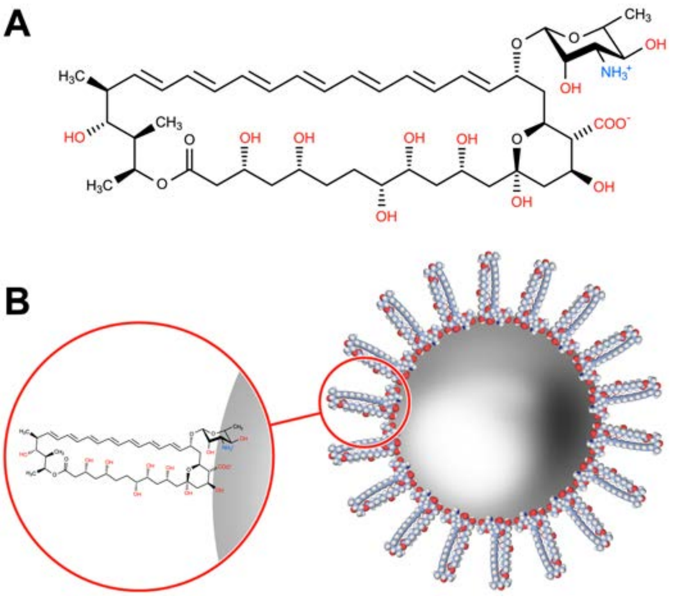

Figure S1. Chemical structure of amphotericin B (panel A) and schematic drawing of the AmB-Ag nanoparticle (panel B).

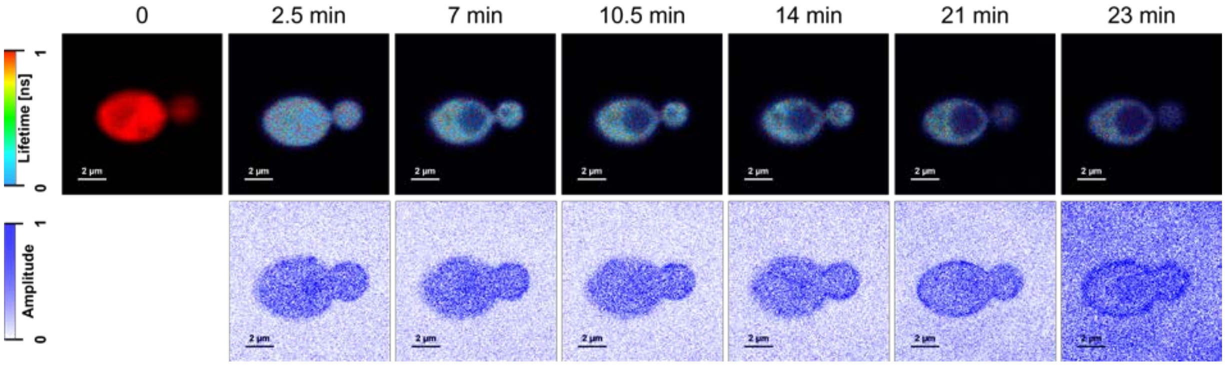

Figure S2. Fluorescence Lifetime Imaging Microscopy images of *C. albicans* cells before (left, 0 min) and after (time in minutes indicated) exposition to AmB-Ag nanoparticles. The upper panels present images based on fluorescence lifetime, below, the same cells are shown imaged based on an amplitude of the short-lifetime fluorescence component (< 300 ps) assigned to AmB. Note the increase of the AmB concentration in the extracellular environment after prolonged incubation. This effect is diagnostic for the disintegration of the cell membrane.

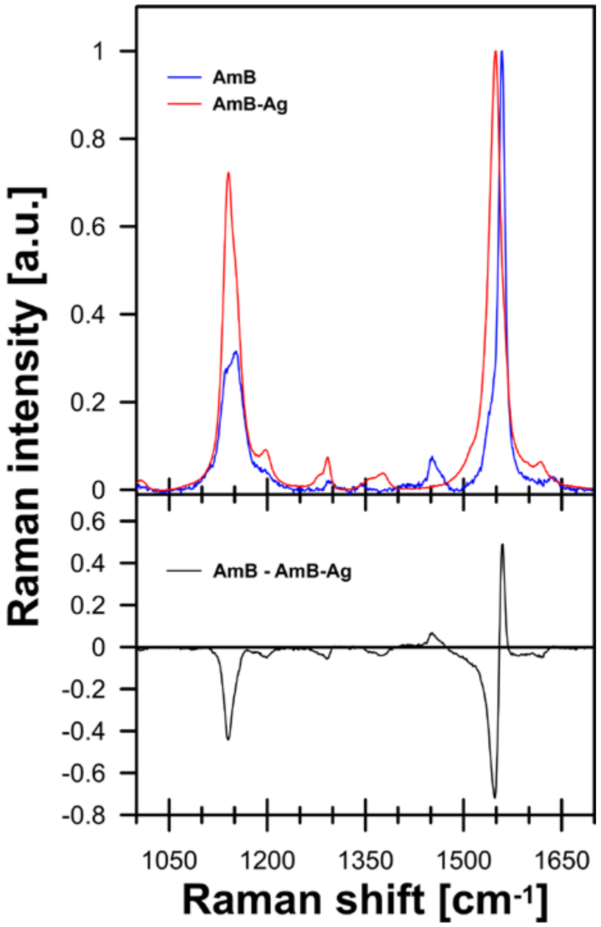

Figure S3. Raman spectra of AmB: recorded directly after the purification with HPLC (blue line) and in the form of hybrid AmB-Ag nanoparticles (red line). Spectra recorded with a 458 nm laser line. The lower panel shows a difference spectrum calculated based on the original spectra displayed in the upper panel.

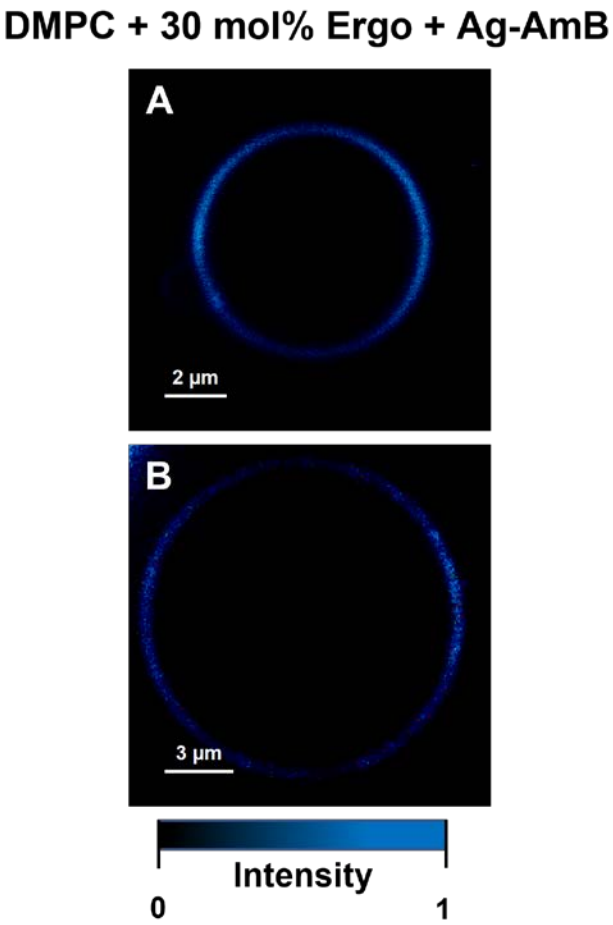

Figure S4. Results of microscopic imaging of a single lipid vesicles composed of DMPC with 30 mol % Ergo with addition amphotericin B. The images are based on fluorescence intensity. The images represent an equatorial vesicle cross-section in the focal plane of the microscope. Maximum fluorescence emission intensities (displayed in blue) on the left and right sides of the liposomes represent the fraction of AmB molecules incorporated perpendicular to the membrane plane (vertical orientation).

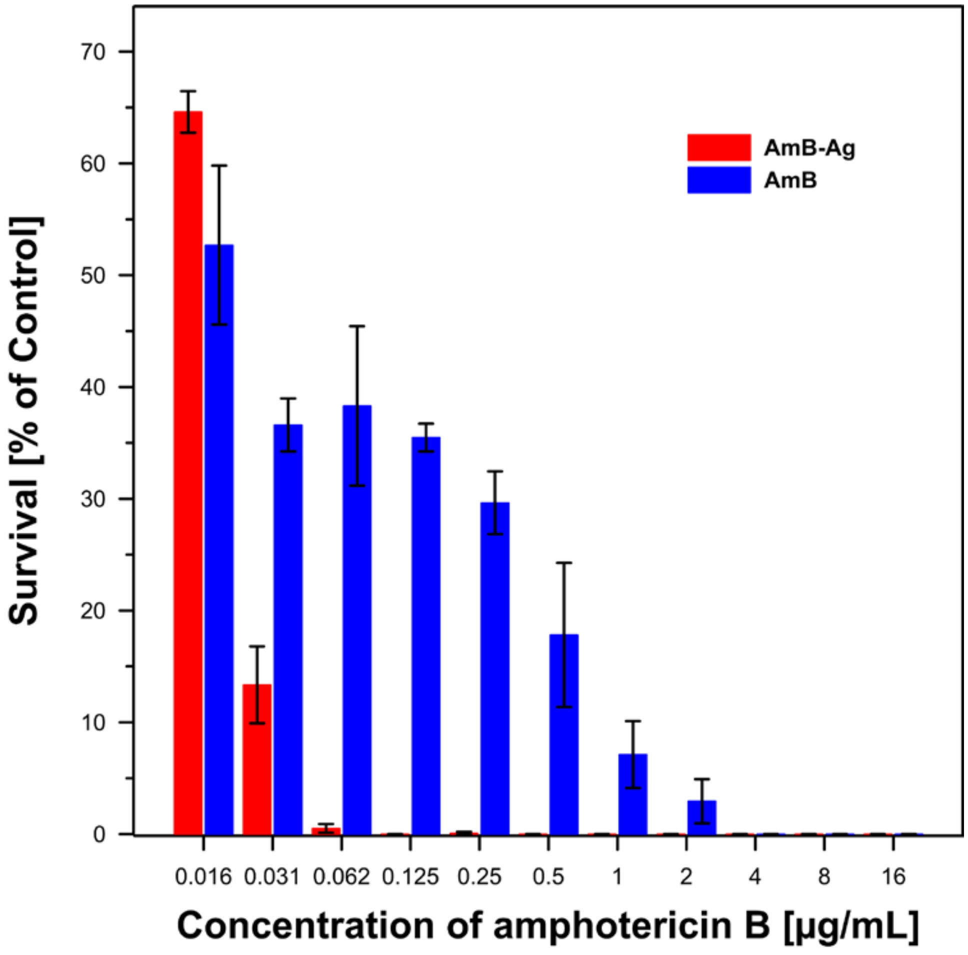

Figure S5. Comparison of the results of *C. albicans* cell viability tests cultured in the presence of AmB and AmB-Ag nanoparticles. Results represent the arithmetic mean ± SD of three independent experiments performed with three replicates for each sample type.
